# Interleukin‐25: New perspective and state‐of‐the‐art in cancer prognosis and treatment approaches

**DOI:** 10.1002/cam4.4060

**Published:** 2021-06-15

**Authors:** Arezoo Gowhari Shabgah, Azwar Amir, Zhanna R. Gardanova, Angelina Olegovna Zekiy, Lakshmi Thangavelu, Maryam Ebrahimi Nik, Majid Ahmadi, Jamshid Gholizadeh Navashenaq

**Affiliations:** ^1^ School of Medicine Bam University of Medical Sciences Bam Iran; ^2^ Student Research Committee Bam University of Medical Sciences Bam Iran; ^3^ Wahidin Sudirohusodo Hospital Makassar Makassar Tamalanrea Indonesia; ^4^ Department of Psychotherapy Pirogov Russian National Research Medical University Moscow Russia; ^5^ Department of Prosthetic Dentistry Sechenov First Moscow State Medical University Moscow Russia; ^6^ Department of Pharmacology, Saveetha Dental College and Hospital, Saveetha Institute of medical and Technical Sciences Saveetha University Chennai India; ^7^ Nanotechnology Research Center, Pharmaceutical Technology Institute Mashhad University of Medical Sciences Mashhad Iran; ^8^ Stem Cell Research Center Tabriz University of Medical Sciences Tabriz Iran; ^9^ Noncommunicable Diseases Research Center Bam University of Medical Sciences Bam Iran

**Keywords:** biomarker, Cancer, cytokine, IL‐17E, IL‐25

## Abstract

Cancer is a leading cause of death which imposes a substantial financial burden. Among the several mechanisms involved in cancer progression, imbalance of immune cell‐derived factors such as cytokines and chemokines plays a central role. IL‐25, as a member of the IL‐17 cytokine subfamily, exerts a paradoxical role in cancer, including tumor supportive and tumor suppressive. Hence, we have tried to clarify the role of IL‐25 and its receptor in tumor progression and cancer prognosis. It has been confirmed that IL‐25 exerts a tumor‐suppressive role through inducing infiltration of eosinophils and B cells into the tumor microenvironment and activating the apoptotic pathways. In contrast, the tumor‐supportive function has been implemented by activating inflammatory cascades, promoting cell cycle, and inducing type‐2 immune responses. Since IL‐25 has been dysregulated in tumor tissues and this dysregulation is involved in cancer development, its examination can be used as a tumor diagnostic and prognostic biomarker. Moreover, IL‐25‐based therapeutic approaches have shown promising results in cancer inhibition. In cancers in which IL‐25 has a tumor‐suppressive function, employing IL‐25‐enhancing approaches, such as Virulizin^®^ and dihydrobenzofuran administration, has potentially inhibited tumor cell growth. On the other hand, in the case of IL‐25‐dependent tumor progression, using IL‐25 blocking methods, including anti‐IL‐25 antibodies, might be a complementary approach to the other anticancer agent. Collectively, it is hoped, IL‐25 might be a promising target in cancer treatment.

## Graphical abstract

This study is focused on the role of IL‐25 in cancer development. It has been shown that IL‐25 exerts conflicting functions in the tumor microenvironment. Using IL‐25‐based therapies might introduce novel approaches in cancer treatment.

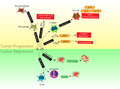

## 1 INTRODUCTION

Cytokines are small molecular proteins (~5–20 kilodaltons) that exert wide‐ranging biological functions.^1^ The immune cells (e.g., macrophages, monocytes, natural killer (NK) cells), B cells, and T cells) and non‐immune cells (e.g., fibroblasts, endothelial cells, and epithelial cells) synthesize and secrete them.^1^ Based on structural similarities, cytokines are divided into several families. Cytokines are able to regulate a variety of biological functions such as innate and adaptive immune responses, cell growth, blood‐cell production, pluripotent stem cell functions, and tissue renewal. Therefore, they are vital in health and disease so that their dysregulation can predispose to inflammation, infection, and cancer.^2^, ^3^ The expression of cytokines affects functions of immune and non‐immune cells in several organs and tissues, especially in the tumor microenvironment.^4^ Tumor cells, tumor stromal cells, and immune cells can express and produce cytokines and their receptors in the tumor microenvironment. Therefore, this process results in alteration in the trafficking and function of various non‐immune and immune cells in the tumor microenvironment. Collectively these alterations influence immune responses and tumor cell behaviors.^4^, ^5^

A large family of cytokines called “cysteine‐knot type cytokines” has a structural motif containing three disulfide bridges stemmed from pairs of cysteine residues.^6^ This group comprises nerve growth factor (NGF), transforming growth factor (TGF)‐β family, platelet‐derived growth factor (PDGF), and interleukin (IL)‐17 family, which has attracted much interest for being involved in regulation of tumor development, invasion, and metastasis.^6^, ^7^, ^8^, ^9^ IL‐17 family, as a recently emerged cytokine subset, exhibited a broad impact on the pathogenesis of various inflammatory diseases. IL‐17 family is composed of six members, including IL‐17A to IL‐17F. IL‐17A (also known as IL‐17) is an important member of this family, which is considered a hallmark of this family and is released by TH17 (T helper 17) cells. IL‐17A shares 16% to 50% similarity with other members, in which IL‐17E (also named IL‐25) has the least resemblance to IL‐17A.^6^, ^10^ Since IL‐25 is the distinguished member of this family, it is suggested that this cytokine might exhibit different functions compared with other members in various biological and pathological conditions, especially in cancer. Thus, in this study, we aimed to discuss the multifarious role of IL‐25 in cancer development.

## 2 IL‐25

### 2.1 Genetic characterization and cell distribution

As mentioned earlier, IL‐25 belongs to the IL‐17 cytokine family, which was initially introduced by Lee et al. in 2001.^11^ According to NCBI GENE (Gene ID: 64806, updated on April 6, 2021), the human *IL25* gene is located on the q‐arm of chromosome 14 (14q11.2) and has two exons. According to UNIPROT (Accession No: Q9H293, updated on April 7, 2021, version 150), this gene encodes a 177‐amino‐acid protein (sequence 1–32 considered a signal peptide). Due to two different alternative splicing, *IL25* mRNA produces two isoforms: subtype 1 is the complete form, which has been cited earlier, while subtype 2 encodes 166 amino acids in which the sequence 1–16 is considered as a signal peptide. In contrast, murine *Il25* is located on chromosome 7 and encodes 169 amino acids which has 80% sequence homology with the human *IL25*. Both human and murine IL‐25 have a conserved cysteine sequence and a potential N‐glycosylation site.^12^ IL‐25 seems to be a unique pleiotropic cytokine that is mainly expressed by helper T cells (TH). In addition to TH cells, it has been shown that dendritic cells (DCs), endothelial cells, epithelial cells, basophils, eosinophils, macrophages, and mast cells also produce this cytokine.^6^

### 2.2 IL‐25 receptor and signaling pathways

There are five proposed receptors for the IL‐17 family, including IL‐17RA to IL‐17RE.^6^ Regarding the receptor, it has been found that IL‐25 is able to bind to IL‐17BR (also called IL‐17Rh1 or IL‐17RB). In addition, it has been confirmed that IL‐25 binding to IL‐17RA is also required for signaling, so that genetic deficiency of either IL‐17RA or IL‐17RB resulted in blockade of IL‐25‐mediated responses.^13^ Therefore, the proposed receptor for IL‐25 is a heterodimer composed of IL‐17RB/IL‐17RA.^13^ It is worth mentioning that in the absence of IL‐25, IL‐17B is also able to interact with IL‐17RB and might trigger signal transduction in the target cells. Therefore, IL‐17B might be responsible for many pathophysiological functions related to IL‐17RB, and IL‐25 might not be involved in these conditions.^14^, ^15^

**FIGURE 1:**
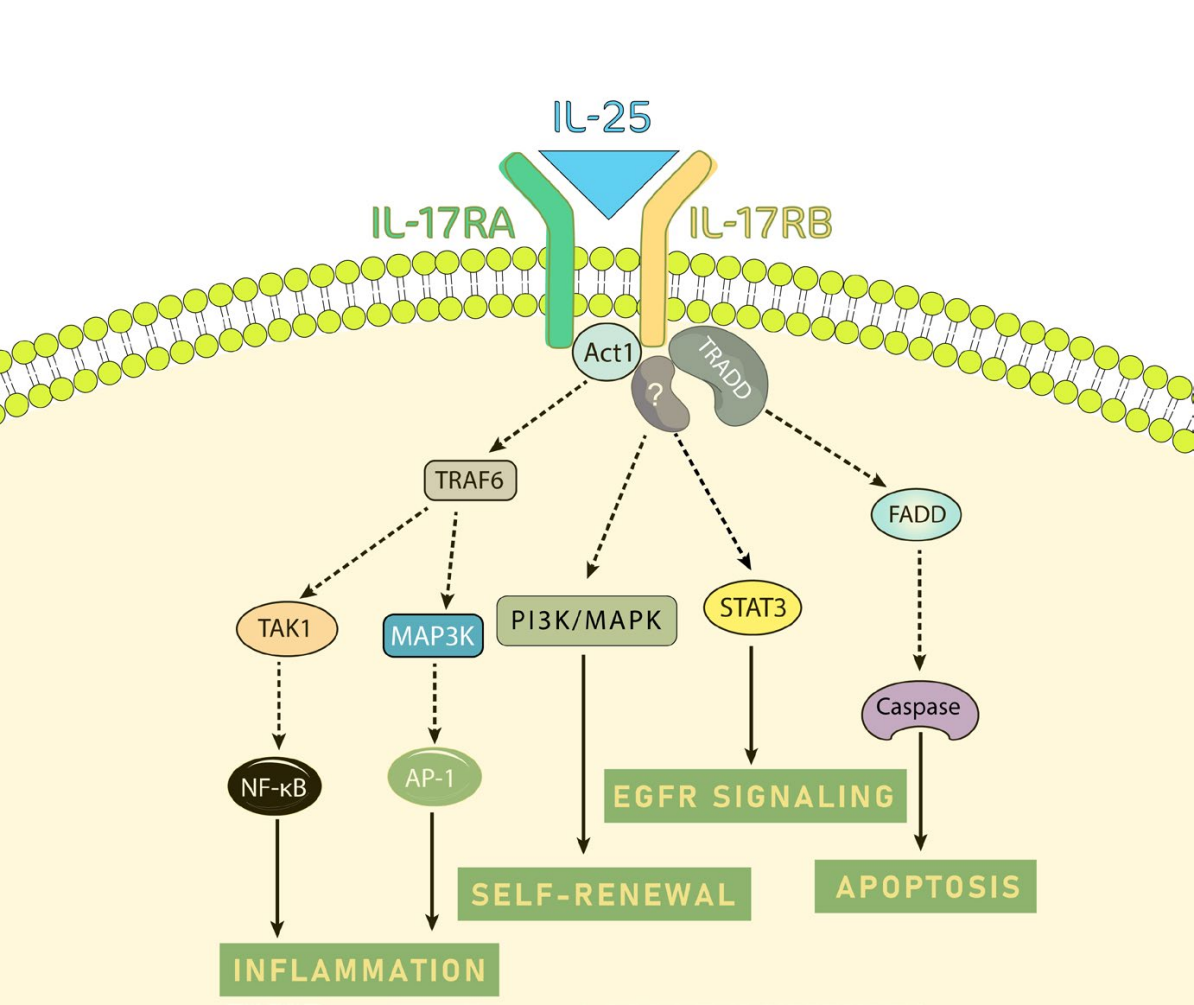
Signaling pathways involved in IL‐25/IL‐25R signaling. IL‐25 ligation to IL‐17RA/IL‐17RB complex recruits adaptor proteins such as Act1, which mediates signal transduction. Act1 employs TRAF6 protein to induce inflammation via transcription factors NF‐κB and AP‐1. TRADD is another recruited adaptor protein to IL‐25R complex, which mediates apoptosis. Also, this receptor complex mediates cancer cell stemness by inducing MAPK/PI3K pathway. Moreover, IL‐25R overlaps with EGFR signaling pathway via activating JAK2/STAT3

The functional receptor IL‐17RB was shown to be expressed on natural killer T cells (NKT), monocytes, L (non‐T/non‐B) cells, nuocytes, innate type‐2 helper (ih2) cells, multipotent progenitor cells, and type‐2 cytokine‐producing myeloid (T2 M) cells. Both IL‐17RA and IL‐17RB sequences contain an intracellular conserved “SEF and IL‐17R” (SEFIR) domain. Having the SEFIR domain confer a capability on these receptors to bind to the proteins that have SEFIR‐containing domains. In other words, SEFIR‐containing proteins are able to interact with each other. Upon IL‐25 ligation to the receptor complex, IL‐17RA/IL17RB heterodimer attracts SEFIR‐containing proteins such as Act1 (a nuclear factor (NF)‐κB activator; also called CIKS) (Figure 1). Act1 is a common signal adaptor protein in the IL‐17R family. Act1 recruits TRAF6 (tumor necrosis factor receptor (TNFR)‐associated factor 6) adaptor, which finally initiates the TAK1 and MAP3K (mitogen‐activated protein kinase) signaling pathways to activate NF‐κB and AP‐1 downstream proteins. Furthermore, IL‐17RB alternatively recruits FADD (Fas‐associated death domain) and TRADD (TNFR1‐associated death domain protein) adaptor proteins, which subsequently activates caspases and apoptosis in the target cells.^6^ Moreover, this receptor complex can also employ other signaling pathways such as JAK/STAT and MAPK/PI3K, indicating a signaling overlap and a synergy with other receptors, including epidermal growth factor receptor (EGFR), which is discussed in the following.^16^, ^17^

### 2.3 Biological activity

The observations regarding the function of IL‐25 have shown that this cytokine skews immune responses toward type‐2 reactions. Therefore, ligation of IL‐25 on TH cells leads to polarization of these cells to TH2 phenotype and upregulation of IL‐4, IL‐5, and IL‐13 cytokines. Moreover, IL‐25 inhibits TH1 and TH17 responses through inhibition of IL‐12 and IL‐23, respectively.^18^, ^19^, ^20^ In this connection, IL‐25‐knockout mice develop EAE (experimental autoimmune encephalomyelitis) and rapid deterioration.^21^ Generally, studies regarding IL‐25 functions have mainly focused on autoimmune diseases, allergic disorders, and parasitic infections.^12^ However, there are pieces of evidence emphasizing the involvement of IL‐25 in cancer development and progression. Therefore, we have decided to shed more light on the role of IL‐25 in cancer.

## 3 IL‐25 EXPRESSION IN CANCER

### 3.1 Dysregulation of IL‐25 and its receptor in serum and peripheral blood cells

Peripheral blood mononuclear cells (PBMCs), which comprise monocytes, T, B, and NK cells, continuously circulates within the blood and multiple tissues, especially in the tumor microenvironment. Therefore, examining these cells provides an opportunity to follow a disease situation. Garley et al. have analyzed the expression and protein level of IL‐25 in neutrophils, PBMCs, and serum. They have revealed that the level of IL‐25 was higher in serum and cell supernatants of the patients suffering from oral epithelial squamous cell carcinoma (OSCC) compared with the healthy subjects.^22^, ^23^ Analysis of IL‐25 expression in PBMCs of malignant breast cancer patients showed that the level of IL‐25 was lower compared with benign and healthy donors. In contrast, IL‐25 receptor expression in PBMC of malignant patients was higher compared with healthy and benign individuals. The secretory and plasma level of IL‐25 in these groups did not differ significantly.^24^ In multiple myeloma (MM), the serum level of IL‐25 also increased compared with healthy subjects, which was positively correlated to the stage of MM. However, the level of this cytokine in MM patients with stage III was decreased and negatively correlated to the cancer stage.^25^

### 3.2 Dysregulation of IL‐25 and its receptor in cancer tissue and tumor cells

In addition to blood components, IL‐25 and IL‐25R (IL‐17RB) expression have also been examined in different types of cancer cell lines and tumor tissues. The RT‐qPCR analysis has shown that IL‐25R expression is significantly higher in malignant cells compared to non‐malignant cells such as MCF‐10A cells. Higher levels of IL‐25R in breast cancer cells proposed that expression of the receptor may endow a higher growth rate to these cells.^26^ In estrogen receptor (ER)‐positive breast cancer cells, Shuai et al. have shown that upregulation of ER resulted in an increased expression of IL‐25 in the tumor cells, and expression of IL‐25 in these cells has led to tumor inhibition.^27^ In B‐cell chronic lymphocytic leukemia (B‐CLL), lower expression of IL‐25 in neutrophils and B lymphocytes has also been observed. In addition, neoplastic cells from B‐CLL reduced the expression of IL‐17RB.^28^ Similar results have been reported by Huang et al. regarding IL‐25 expression in non‐small‐cell lung cancer (NSCLC).^29^ In aggressive grades of prostate cancer, it has also been shown that the level of IL‐25 was decreased. In this regard, expression of IL‐25 and IL‐25R in prostate cancer tissue samples of prostate cancer reversely correlated to Gleason grade, suggesting that IL‐25 might be involved in the pathogenesis.^30^

In colorectal cancer (CRC), analyzing the expression pattern of IL‐25 via immunohistochemistry has shown that this cytokine is strongly expressed in both healthy subjects and patients without any significant difference. Also, expression of IL‐17RA/IL‐17RB heterodimer remained unchanged between healthy and colorectal cancer patients so that the receptor complex in the colorectal epithelium of both groups was abundantly expressed.^31^ Although expression of IL‐25 and IL‐25R have not differed between healthy subjects and cancer patients, Thelen et al. have shown that blockade of IL‐25 in colitis increased the risk of tumor progression and tumor burden. These results propose that IL‐25 might play a suppressive function in development and growth of colonic tumors.^32^ In another study, Lui et al. have found that IL‐25 and its receptor were simultaneously decreased in CRC compared with ulcerative colitis.^33^ Therefore, in inflammatory conditions, such as colitis, the production of IL‐25 and IL‐25R might be favorable for CRC inhibition.^32^, ^33^

## 4 THE ROLE OF IL‐25 IN CANCER

**FIGURE 2:**
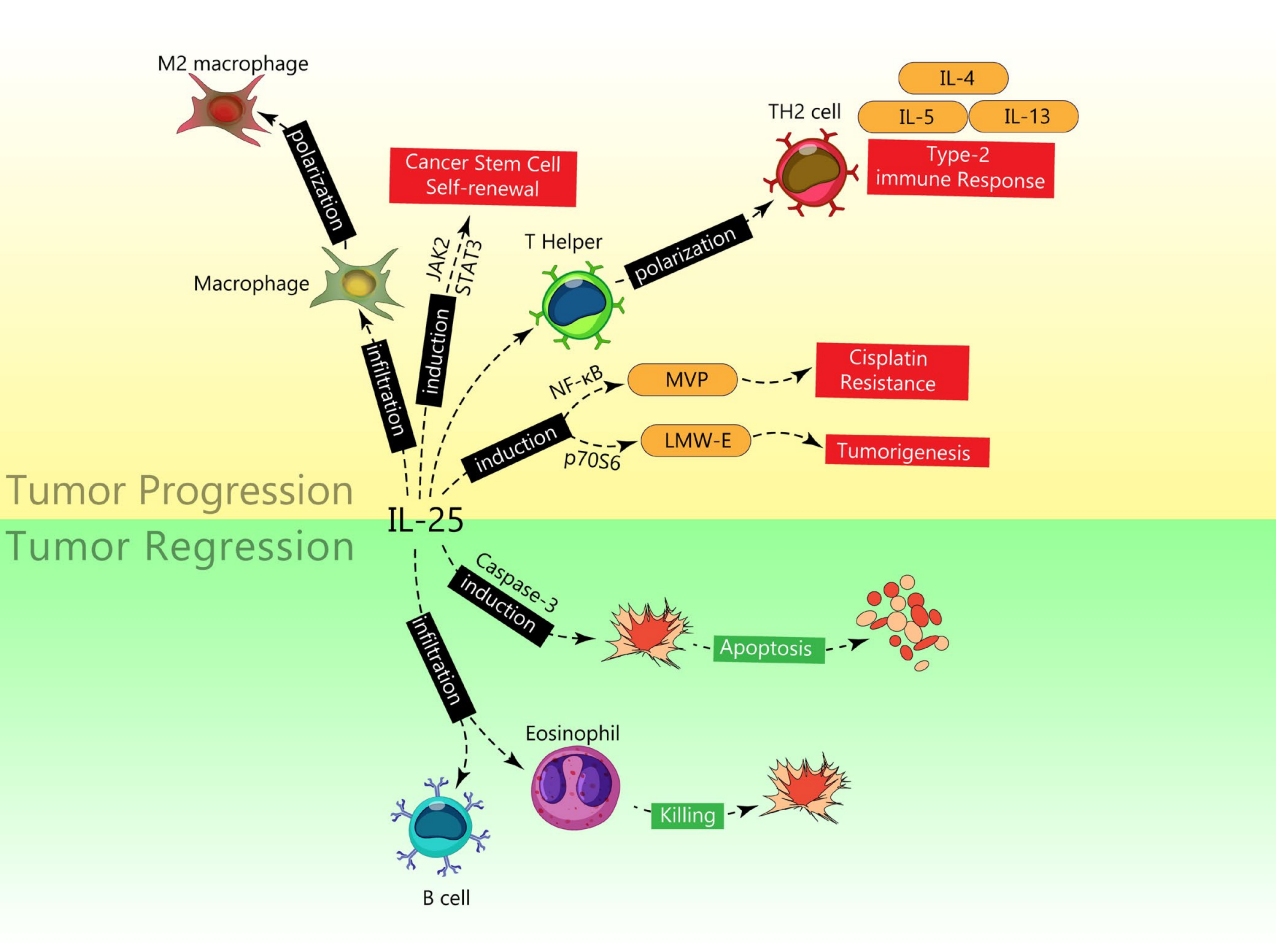
Tumor‐supportive and tumor‐suppressive roles of IL‐25 in cancer. In tumor‐supportive function (shown in the yellow area), IL‐25 binding to its receptor on macrophages and TH cells mediates their conversion into M2 macrophages and TH2 cells, respectively. Also, IL‐25 via induction of JAK2/STAT3 signaling promotes cancer stem cell's self‐renewal. Moreover, induction of MVP and LMW‐E via IL‐25 leads to tumorigenesis and cisplatin resistance. In tumor‐suppressive function (shown in the green area), IL‐25 activates caspase enzymes to induce apoptosis in tumor cells. Also, IL‐25 infiltrates eosinophils and B cells into the tumor microenvironment to mediate tumor cell killing

The process of cancer development has not yet been fully understood, and involvement of several factors has been attributed to cancer initiation. Inflammation has been proposed as one of the initiators.^34^ During cancer progression, inflammatory cells are recruited into the tumor microenvironment by several mediators such as secretion of cytokines and chemokines, hypoxia, and oncogenic changes. Inflammatory cells in the tumor microenvironment are comprised of recruited immune cells and activated myofibroblasts. These cells secrete growth factors, cytokines, and chemokines.^35^ Among secreted factors, cytokines modulate inflammation in the tumor microenvironment. Previous studies indicated that cytokines including TNF‐α, IL‐1, IL‐6, and TGF‐β are integral parts of the tumor microenvironment.^36^ Concerning the IL‐17 family, especially IL‐25, it has been shown that this cytokine has been dysregulated during cancer (Table 1). Therefore, recent investigations have indicated that the IL‐17 family exerts pivotal roles in cancer.^37^ Several studies have confirmed that IL‐25 exerts a tumor‐supportive function, while a few studies have also conveyed that IL‐25 may act as a tumor‐suppressive factor (Figure 2).^38^ The tumor‐supportive roles of IL‐25, which in this regard is mainly produced by CAFs, have been shown to promote cell cycle, induce EMT and metastasis.^39^ In contrast, IL‐25 ligation to IL‐25R on tumor cells mediates antitumor effects through induction of apoptosis and infiltration of eosinophils and B cells.^26^, ^40^

**TABLE 1:** The diverse role of IL‐25 in various cancer

Cancer Type	IL−25 expression in tumor cells compared with normal tissues or cells	Role in Cancer	Prognosis	Study Model	References
Colorectal Cancer	Highly expressed in both healthy and CRC patients	Not defined	Not defined	Histopathological samples	[31]
	Highly expressed in colon polyp and ulcerative colitis than CRC	Not defined	Not defined	Histopathological samples	[33]
	Downregulated compared with colitis	Tumor suppressive	potential protective marker	Murine model	[32]
Cervical Cancer	Overexpressed	Tumor supportive	Potential negative prognostic factor	HeLa and SiHa cell lines	[41]
Gastric Cancer	Highly expressed	Tumor suppressive	Favorable prognosis after resection	Histopathological samples	[43]
Lung Cancer	Overexpressed in cisplatin‐resistance cells	Tumor supportive	Not‐defined	A549 cell line	[38]
Multiple Myeloma	Highly overexpressed	Not defined	Positively correlated to disease stage	Serum samples	[25]
Cutaneous T Cell Lymphoma	Overexpressed	Tumor supportive	Not defined	Histopathological samples	[44]
B‐cell Chronic Lymphocytic Leukemia	Reduced expression in PMN and B cells and elevated in sera at stage 0/I and II	Not defined	Not defined	PMNs, B lymphocytes, and serum	[28]
Oral Epithelial Squamous Cell Carcinoma	No significance difference in PBMCs and PMNs but higher level in cell supernatants and blood serum	Tumor supportive	Not defined	PMNs, PBMCs, and serum	[22]
Hepatocellular Carcinoma	Overexpressed in cancer stem cells	Tumor supportive	Not defined	Huh7 and PLC/PRF/5 cell lines murine model	[17]
Cholangiocarcinoma	Overexpressed	Not defined	Poor prognosis	Histopathological samples	[55]
Prostate Cancer	Overexpressed in prostate cancer and benign prostatic hyperplasia	Tumor supportive	Not defined	Histopathological samples	[30]
Bladder Cancer	Elevated in bladder polyp and cystitis compared with bladder cancer	Tumor suppressive	Not defined	Histopathological samples	[57]
Breast Cancer	Q2‐3 induced its expression	Tumor suppressive	Not defined	4T1 and MDA‐MD−231 cell lines/animal study	[39]
	Upregulated in tumor cells	Tumor supportive	Not defined	MCF−7, T47D cell lines and ER^–^ breast cancer clinical samples	[46]
	Upregulated during mammary gland's development but noticeably decreased in mature mouse and human mammary glands	tumor suppressive	Not defined	MCF−7, MDA‐MB468, SKBR3, T47D, and MCF−10A cell lines /murine model	[26]
	Increased in ER^+^ than ER^–^ breast cancer	Tumor suppressive	Potential good prognostic factor	TCGA of breast cancer dataset	[27]
	Downregulated in non‐malignant and further in malignant samples	Not defined	Negative correlation with cancer grade	PBMCs and serum	[24]

Abbreviations: CRC, colorectal cancer; ER, estrogen receptor; PBMCs, peripheral blood mononuclear cells; PMNs, polymorphonuclears; TCGA, The Cancer Genome Atlas.

### 4.1 Mechanisms of IL‐25‐dependent tumor progression

The function of IL‐25 in cancers is primarily tumor‐supportive. To address this issue, Cheng et al. have shown that expression of IL‐25 and IL‐25R were upregulated in HeLa and SiHa cells (cervical cancer cell lines).^41^ Furthermore, it has been indicated that administration of recombinant IL‐25 increases the viability, invasion, and migration of tumor cells, while blockade of IL‐25 with neutralizing antibodies leads to the apoptosis of tumor cells.^41^

#### 4.1.1 The role of IL‐25 in cell resistance to apoptosis and cancer stemness

Lung cancer is one of the cancers in which IL‐25 plays a supportive role in its development. In this regard, it has been shown that IL‐25 was associated with cisplatin resistance in the A549 cell line, thereby downregulation of IL‐25 in these cells reversed drug resistance. Major vault protein (MVP) has been proposed as a target gene in this case, which mediates cisplatin resistance. Shen et al. have found that overexpression of IL‐25 enhances NF‐κB activity and consequently upregulates MVP expression in lung cancer cells.^38^ Non‐cancer cell stem cells (non‐CSCs) play a pivotal role in hepatocellular carcinoma (HCC) progression. The cross‐talk between non‐CSCs and CSCs leads to cancer stemness. Luo et al. have found that IL‐25/IL‐17RB mediates this cross‐talk. Non‐CSCs secrete IL‐25 into the tumor microenvironment, and secreted IL‐25 interacts with IL‐17RB on CSCs. On the one hand, this interaction activates the JAK2/STAT3 signaling pathway in CSCs, leading to CSC self‐renewal. On the other hand, IL‐25/IL‐17RB interaction activates the NF‐κB signaling pathway, which results in proliferation and apoptosis resistance in CSCs.^17^ Therefore, non‐CSCs sustain CSCs in the tumor microenvironment through secretion of IL‐25 and contribute to cancer stemness.^17^

#### 4.1.2 The role of IL‐25 in skewness of immune responses in favor of tumor promotion

As previously mentioned, IL‐25 skews TH differentiation towards TH2, and TH2 cells mainly promote tumor progression. In a mouse model of highly malignant spontaneous breast tumor called MMTV‐PyMT transgenic mouse, tumor‐infiltrating CD4^+^ TH2 cells highly express IL‐25R, while CD8^+^ T cells don't express this receptor. These TH2 cells produce IL‐4, facilitate macrophage conversion into M2 type, and inhibit cytotoxic T cells (CTLs) responses, resulting in tumor metastasis.^42^ In gastric cancer (GC) patients, it has been demonstrated that TGF‐β1 dose‐dependently induces tumor‐resident macrophages to produce IL‐25, which consequently promotes GC.^43^

The elevated level of IL‐25 in cutaneous T‐cell lymphoma (CTCL) patients has also been observed. In this case, keratinocytes are the main secretors of IL‐25. The secretion of IL‐25 into the epiderm activates IL‐25R‐expressing T cells, which enhances skewness of TH cells toward TH2 lymphocytes through phosphorylation of STAT6. IL‐25‐associated polarized TH2 cells secrete IL‐4, IL‐5, and IL‐13 to augment and create a more TH2‐dominant microenvironment. This microenvironment inhibits antitumor TH1 responses and promotes CTCL.^44^ In another study by Liu et al., it has been shown that IL‐25 promotes HCC progression and metastasis via enhancement of M2 macrophage polarization. Further evaluations have found that gut microbiota dysbiosis resulted from antibiotic treatment is responsible for hyperplasia of tuft cells (the epithelial lining of the intestines) to produce IL‐25 in a murine model. Tuft cell‐derived IL‐25 induces M2 macrophages to produce chemokine CXCL10, which in turn mediates migration of HCC cells via upregulation of Snail, vimentin, and phosphorylation of ERK. Finally, this process promotes epithelial‐mesenchymal transition (EMT).^45^

#### 4.1.3 The role of IL‐25 in promoting cell cycle

Mobelli et al. have indicated that pro‐tumoral activity of IL‐25 in breast cancer could be attributed to overexpression of low molecular form of cyclin E (LMW‐E). The formation of LMW‐E is considered a poor disease‐free survival factor in breast cancers, and it is generated by proteolysis from the full‐length cyclin E, resulting in a missed regulatory domain of cyclin‐E. In this regard, they have shown that IL‐25 and IL‐17RB interaction phosphorylates c‐Raf, ERK, and p70S6 kinases in breast cancer cells (MCF‐7, IJG‐1731, and T47D cells lines). Activation of this pathway leads to the generation of LMW‐E rather than cyclin‐E (a regulatory subunit of cyclin‐dependent kinase 2 (Cdk2)) that contributes to G_1_/S transition. The expression of LMW‐E endows tumorigenic phenotype to breast cancer cells. Furthermore, docetaxel resistance also results from this process which is initiated by IL‐25/IL‐17RB signaling.^46^

#### 4.1.4 Synergy of IL‐25 signaling with other tumor‐promoting growth factors

Triple‐negative breast cancer (TNBC) is considered a highly aggressive phenotype among breast tumors. Signaling of epidermal growth factor receptor (EGFR) in TNBC, which activates Src kinase activation, is one of the major causalities of tumor progression. TNBC is treated chiefly with anti‐EGFR blocking antibodies and tyrosine kinase inhibitors (e.g., IRESSA^®^, also called gefitinib). Merrouche et al. have shown that IL‐25R signaling Src kinase‐dependently phosphorylates EGFR and overlaps with EGFR signal transduction, which can be inhibited by AZM475271 (an Src kinase‐specific inhibitor) in IRESSA^®^‐resistant TNBC cells (MDA‐MB468 and BT20 cell lines). This process implies a synergy between IL‐25R and EGFR signal transduction, which finally mediates STAT3 activation and tumor development.^16^

### 4.2 Mechanisms of IL‐25‐associated tumor suppression

In addition to the tumor‐supportive role of IL‐25, it has been clarified that this cytokine can also exert tumor‐suppressive function. It has been shown that IL‐25 induces IL‐5 production from TH2 cells. IL‐5 secretion in tumor microenvironment leads to activation of CCR3 (CC motif chemokine receptor type 3)‐expressing eosinophils in blood and spleen of tumor‐bearing mice. The expression of this receptor gives rise to infiltration of activated eosinophils into tumor nest to exert antitumor activity. In addition to eosinophil, it has been demonstrated that B cells are also required to fulfill IL‐25’s antitumor function in vivo. In this regard, it has been shown that IL‐25 activates NF‐κB signaling through phosphorylation of the IκB‐α subunit (inhibitory subunit of NF‐κB).^47^ Of note, tumor‐infiltrating B cells were widely investigated in breast tumors, where they comprise up to 40% of tumor‐infiltrating lymphocytes (TILs) in about 25% of tumors.^48^ Collectively, the activation of eosinophils and B cells leads to fighting against progression of colon adenocarcinoma, NSCLC, melanoma, breast cancer, and pancreatic cancer.^47^

Ligation of IL‐25 to its receptor, IL‐17RB, on breast cancer cells also induces apoptosis. As has been mentioned, IL‐17RB is differently expressed in breast tumor cell lines. The interaction of IL‐25 with its receptor induces death signals in breast cancer cells in vitro. It has been found that there is a region in the intracellular region of IL‐25R which shares about 30% similarity with death domains (DDs). Upon IL‐25 ligation to highly IL‐25R‐expressing tumor cell lines, DD‐containing proteins, including TRADD and FADD, are recruited to the receptor complex. Recruitment of these proteins initiates cell death signaling, which subsequently induces caspase‐mediated apoptosis.^26^

## 5 IL‐25 AS A TUMOR BIOMARKER

A tumor biomarker refers to a substance or product that is indicative of tumor presence in the body. A biomarker can be a tumor‐secreted molecule or particular reaction to the presence of cancer in the body.^49^ Recently, the upregulation or downregulation of cytokines, chemokines, and growth factors have been attracted much attention to be identified as biomarkers.^50^, ^51^ Therefore, examining these factors might be helpful for cancer diagnosis and prognosis.^52^, ^53^, ^54^ In the Table 1, it was listed various cancers in which IL‐25 might be a potential biomarker in tumor prognosis and diagnosis.

### 5.1 Hepatocellular carcinoma and cholangiocarcinoma

As has been noted in the former sections, IL‐25 is dysregulated in several cancers. The analysis of IL‐25 expression patterns in various tissues of cancer patients might imply the tumor presence in the body. Kaewsarabhumi et al. have confirmed that higher expression of IL‐25 can be a diagnostic marker in cholangiocarcinoma. They have shown that expression of IL‐25 was significantly higher in metastatic cholangiocarcinoma compared with non‐metastatic cholangiocarcinoma patients. Moreover, survival analysis discovered that upregulation of IL‐25 was associated with shorter survival time in cholangiocarcinoma patients.^55^ Further analysis regarding IL‐25 involvement in cholangiocarcinoma using protein interaction software STITCH has shown that IL‐25 has potential direct and indirect interaction with SMAD2, TGF‐β1, NF‐κB, and SNAI. It is proposed that SMAD2 and TGF‐β1 expression might be involved in IL‐25 elevation and EMT. Although these interactions have not been verified experimentally, it is proposed that IL‐25 is associated with these factors to mediate EMT in cholangiocarcinoma.^55^

In HCC, it has been found that IL‐25 was significantly increased in the tissue and serum of patients, and elevated IL‐25 expression in the HCC tissue was negatively associated with survival rate and correlated to poor prognosis after radical hepatectomy.^45^ Regarding IL‐25 involvement in HCC progression, it has been proposed that this cytokine facilitates tumorigenesis via inducing M2 macrophage development through induction of ERK signaling in these cells. Mechanistically, HCC cells upregulate mesenchymal markers, including Snail and vimentin, and downregulate epithelial markers such as E‐cadherin when co‐cultured with IL‐25‐induced M2 macrophages. Also, IL‐25‐induced M2 macrophages secreted CXCL10 to induce migration of HCC cells. These data imply IL‐25‐dependent HCC progression.^45^

### 5.2 Prostate cancer

Prostate cancer (PCa) and its non‐metastatic form called benign prostatic hyperplasia (BPH) are considered the most frequent urological diseases in aged men. The risk of PCa development from BPH is one of the healthcare problems among the urological community. Therefore, defining a suitable biomarker to evaluate BPH conversion to PCa is crucial. It has been shown that IL‐25 expression is elevated in both BPH and PCa, but its level is inversely associated with Gleason grade in PCa. In contrast, results showed that IL‐25R expression in PCa was reduced compared to BPH. Although there is no correlation between BPH progression to PCa and IL‐25 or IL‐25R expression, these two molecules might be helpful for PCa grading.^30^ Also, it is suggested that these two factors might be complementary biomarkers to other established PCa markers such as prostate‐specific antigens (PSA) to increase their specificity and sensitivity. Although there is no defined underlying mechanism concerning IL‐25’s role in prostate cancer, it is proposed that IL‐25 interaction with IL‐25R might exert an antitumor function.^30^

### 5.3 Breast cancer

Analysis of IL‐25 expression in malignant breast cancer patient‐derived PBMCs showed that the level of IL‐25 was lower compared with benign and healthy donors. In contrast, IL‐25 receptor expression in PBMC of malignant patients was higher compared with samples of healthy and benign individuals. The secretory and plasma levels of IL‐25 in these groups did not differ significantly.^24^ In addition to peripheral blood, it has also been stated that expression of IL‐25R in breast tumor tissues correlated to cancer aggressiveness and poor prognosis. Huang et al. have affirmed that the amplified expression of IL‐25R is linked to breast tumorigenesis and may serve as a better prognostic marker compared to HER2 amplification so that IL‐25R expression correlates to poor prognosis compared to HER2^+^ breast cancer. In this regard, it has been shown that IL‐17B binding to IL‐25R (IL‐17RB) in the absence of IL‐25 activates NF‐κB signaling by enhancing TRAF6 and exerts an antiapoptotic role via Bcl‐2 upregulation.^56^

### 5.4 Other cancers

In addition to prostate, as mentioned earlier, discrimination of inflammatory conditions from tumor microenvironment might be achieved through comparing the expression of IL‐25 in these two different microenvironments. For instance, IL‐25 and IL‐25R expression in inflammatory conditions such as bladder polyp or ulcerative colitis are increased compared to bladder cancer or CRC, respectively. However, the underlying mechanism involved in the contribution of IL‐25 and IL‐25R in colorectal and bladder cancer regression has not yet been defined. Therefore, using these two markers might be helpful for prognosis and diagnosis.^33^, ^57^ In gastric cancer, Kaplan–Meier survival analysis demonstrated a positive correlation between the density of intra‐tumoral IL‐25^+^ macrophages and 5‐year or overall survival in patients. Therefore, it has been revealed that density of intra‐tumoral IL‐25^+^ macrophages, TNM stage, and tumor location were independent predictive biomarkers for overall survival in patients who have gastric cancer. Intratumoral IL‐25^+^ macrophages are proposed to regulate the ratio of effector T cells/regulatory T cells. Moreover, IL‐25^+^ macrophages positively correlated to the elevation of CD8^+^ T cells and negatively related to infiltration of T reg cells. Taking together, these results showed that IL‐25^+^ macrophages, which inhibited the tumor progression, were a predictor biomarker of favorable survival in gastric cancer patients after resection.^43^

## 6 THERAPEUTIC APPROACHES

Since Benatar et al. study has shown that IL‐25 exerts antitumor function through activation of eosinophils and B cells, it is concluded that injection of recombinant IL‐25 (rIL‐25) might be effective for tumor inhibition. Therefore, they have shown that administration of rIL‐25 exhibited antitumor effects on pancreatic, lung, colorectal, and breast cancers in xenograft nude mice models and human melanoma in a dose‐dependent manner.^47^ Furthermore, Furuta et al. have shown that highly IL‐25R‐expressing malignant breast tumor cells, upon interaction with rIL‐25, induce apoptosis in these cells. Therefore, they have suggested that using rIL‐25 might be useful to treat IL‐25‐expressing malignant tumor cells.^26^

Virulizin® is an anticancer and immunomodulatory agent, which is prepared from bovine bile. Some preclinical studies have shown that Virulizin® administration inhibited the growth of human xenograft tumor models, including melanoma, ovarian, pancreatic, prostate, and breast cancers. The primary antitumor function of Virulizin® relies on NK cell activation and their infiltration into the tumor microenvironment. Further investigation has indicated that Virulizin® stimulates IL‐25 production predominantly from B cells, resulting in elevated blood eosinophilia and infiltration of activated eosinophils to the melanoma microenvironment.^40^ In addition to Virulizin®, a synthetic agent called dihydrobenzofuran lignan (Q2‐3) exerts antitumor effects on MDA‐MD‐231 and 4T1 breast cancer cells. Yin et al. have shown that Q2‐3 induces fibroblast expansion and their ability to secrete IL‐25. Secretion of IL‐25 from fibroblasts inhibited breast tumor metastasis. Also, Q2‐3‐induces IL‐25 from fibroblasts and activates caspase‐3 and caspase‐8, leading to cell apoptosis. The comparison of docetaxel and Q2‐3 efficacies has shown that Q2‐3 was more effective than docetaxel in inhibition of tumor growth and metastasis.^39^

Combination therapies in cancer have attracted much attention in recent years to avoid drug resistance in target cells. Hence, simultaneous administration of multiple medications diminishes the chance of tumor relapse. A combination of rIL‐25 with IL‐17B silencing (siIL‐17B) and melatonin could vigorously inhibit cancer progression. Gelateli et al. have demonstrated that this combination decreased VEGF expression, diminished cell viability, and encouraged apoptosis in breast cancer cells. Taking together, using IL‐17B inhibitors increases the therapeutic efficacy of rIL‐25 in cancer.^15^

Single‐chain variable fragments (scFv) are fusion proteins that consist of the variable regions of the light and heavy chains of immunoglobulins. These proteins have shown potential therapeutic advantages over whole antibodies. Younesi et al. have introduced two anti‐IL25R scFvs for evaluating the antitumor effects of these agents. They have shown that using anti‐IL‐25 scFvs inhibited tumor growth compared with normal cells. These agents also exhibited antiproliferative and pro‐apoptotic effects, through activation of caspase‐3, on IL‐25R‐expressing breast cancer cell lines, including MCF7 and SKBR3 cells.^58^

As has been discussed, although IL‐25 inhibits tumor growth in some cases, it has been shown that IL‐25 also plays a pivotal role in cancer progression in several studies. Cisplatin, as a potent anticancer drug, is frequently used against several types of solid tumors. This agent remarkably downregulates expression of IL‐25 and IL‐25R and reverses the regulatory effects of rIL‐25 on viability, migration, and invasion of cervical cancer cells. These results propose that cisplatin hinders the viability, migration, and aggressiveness of tumor cells and promotes cancer cell death, possibly by downregulating IL‐25/17RB signaling. Hence, cisplatin might be effective for treating cervical cancer patients with upregulated IL‐25 expression.^41^

In metastatic breast cancer, upregulation of IL‐25 and IL‐17RB has been observed by Jiang et al. They have emphasized that using blocking anti‐IL‐25 antibodies may provide an innovative approach to treat human breast cancer.^42^ Therefore, administration of anti‐IL‐25 neutralizing antibodies decreased the IL‐4‐producing CD4^+^ T cells and M2 macrophages and reduced metastasis of breast cancer cells into lung tissue in the MMTV‐PyMT transgenic mice model. These results resemble the anti‐IL‐4 blocking antibody results in breast tumor treatment.^42^ In TNBC, in which there is an overlap between IL‐25R and EGFR signaling, it is proposed that inhibition of shared adaptor proteins or kinases via drugs, including Src kinase‐specific inhibitors (e.g., AZM475271), might be promising in treatment of aggressive type of breast cancers.^16^

Since IL‐17RB is a common receptor for IL‐25 and IL‐17B, it is found that IL‐17B can induce pro‐neoplastic properties in cancer cells. IL‐17B/IL‐17RB interaction stimulates Bcl‐2 (an antiapoptotic protein) expression to exert an apoptosis‐inhibitory effect through the NF‐κB pathway. Further investigation affirmed that the initiation of IL‐17RB/IL‐17B signaling is crucial for breast tumorigenesis which its expression is correlated with HER2 amplification and poor prognosis in breast cancer patients. Therefore, inhibition of IL‐17RB (IL‐25R) signaling is another therapeutic approach in cancer treatment. In this regard, it has been demonstrated that neutralizing anti‐IL‐25R antibodies suppresses tumorigenicity of breast cells. As such, the depletion of IL‐25R in trastuzumab (anti‐HER2 antibody)‐resistant breast cells can vividly decrease tumorigenesis.^56^

## 7 CONCLUDING REMARKS AND FUTURE DIRECTIONS

IL‐25, as a member of the IL‐17 family, mainly contributed to inflammation and allergic disease. Recently, this cytokine has been attracted much attention in cancer research. IL‐25 is produced not only by immune cells (e.g., T cells, DCs, and macrophages) but also other non‐immune cells (e.g., fibroblasts, epithelial cells, keratinocytes) and even some cancer cells (such as melanoma, liver, breast, and cervical cancers). The expression of IL‐25 and its receptor have been dysregulated in various cancers compared with normal tissues. Therefore, it has been indicated that IL‐25 might play a multifarious role in cancer progression or regression. The tumor‐suppressive role of IL‐25 is mainly attributed to the infiltration of eosinophils and B cells into the tumor microenvironment and induction of apoptosis. In contrast, its tumor‐supportive roles rely on the deviation of immune responses and stimulation of EMT and cell growth. Although a couple of mechanisms related to IL‐25 involvement in tumor development have been discovered, it is necessary to be investigated more detailed underlying mechanisms. Most studies concerning IL‐25 involvement in cancer have been studied *in vitro* and cancer cell lines. Therefore, it is suggested that these investigations should be more focused, at least in animal models. Moreover, since IL‐17B and IL‐25 have a common receptor called IL‐17RB, it is also required to define which ligand is involved in the cancer progression. Also, considering that non‐coding RNAs such as long non‐coding RNAs (lncRNAs) and microRNAs (miRNAs) are involved in protein expression, it is also suggested that contribution of these RNAs in dysregulation of IL‐25 and its receptor should be included in future studies.

IL‐25 holds great promise for developing effective and novel cancer treatment with a broad therapeutic approach. Recent investigations have reported that IL‐25 targeting in IL‐25R‐expressing cancer results in positive clinical responses. Since tyrosine kinases are involved in IL‐25R signaling, using tyrosine kinase inhibitors (TKIs) in cancers in which IL‐25 promotes its progression might provide a new approach in cancer therapies. Aptamers and small IL‐25 inhibitors also should be investigated in this regard. It is hoped that using combination therapies with an IL‐25‐based therapeutic approach might be promising in cancer treatment.

Besides therapeutic approaches, IL‐25 dysregulation in body fluids, peripheral blood cells, and tumor tissue might be a valuable biomarker for predicting cancer prognosis. Regarding using IL‐25 as a biomarker, since immune responses are relatively different at each stage of diseases, this study proposes that future investigations should be more focused on the dysregulation of IL‐25 at different stages of cancer. Also, it is important to determine the sensitivity, specificity, and accuracy of IL‐25 levels in various cancers. In addition, defining the involvement of IL‐17B, which shares the receptor with IL‐25, and analyzing its level accompanied with IL‐25 and IL‐25R is crucial. Finally, we anticipate that our present review study may lead to development of IL‐25‐based diagnosis and prognosis. It is hoped that studies regarding newly identified cytokines such as IL‐25 involvement in cancer would expand our knowledge regarding cancer.

## AUTHOR CONTRIBUTIONS

Arezoo Gowhari Shabgah, Investigation & Methodology; Azwar Amir, Writing – Review & Editing; Zhanna R. Gardanova, Writing – Review & Editing; Angelina Olegovna Zekiy, Writing – Review & Editing; Lakshmi Thangavelu, Writing – Review & Editing; Maryam Ebrahimi Nik, Validation & Investigation; Majid Ahmadi, Methodology & Investigation; Jamshid Gholizadeh Navashenaq, Supervision & Validation.
